# Pyranopyrazole based Schiff base for rapid colorimetric detection of arginine in aqueous and real samples†

**DOI:** 10.1039/d2ra00091a

**Published:** 2022-04-19

**Authors:** Rashim Bawa, Nidhi Deswal, Swati Negi, Manu Dalela, Amit Kumar, Rakesh Kumar

**Affiliations:** Bioorganic Laboratory, Department of Chemistry, University of Delhi Delhi 110007 India rakeshkp@email.com; Stem Cell Facility (Centre of Excellence for Stem Cell Research), All India Institute of Medical Sciences New Delhi 110029 India; Department of Chemistry, Dyal Singh College, University of Delhi Delhi 110003 India

## Abstract

A novel pyranopyrazole-based Schiff base PPS has been synthesized *via* a condensation reaction between aldehyde and hydrazide derivatives of pyranopyrazole. The probe acted as a selective and sensitive chemosensor for the colorimetric detection of arginine under aqueous conditions with a detection limit of 1.8 × 10^−5^ M. The 1 : 1 binding stoichiometry was established using various UV-vis spectroscopic methods. A plausible binding mechanism of PPS towards arginine was established *via*^1^H NMR titration techniques and the results were further validated using DFT studies. Moreover, PPS provided a reasonable response for arginine in dietary supplements and human blood plasma which demonstrates its potential application in real sample analysis as well.

## Abstract 2

A novel pyranopyrazole-based Schiff base PPS has been synthesized *via* a condensation reaction between aldehyde and hydrazide derivatives of pyranopyrazole.

## Graphical abstract

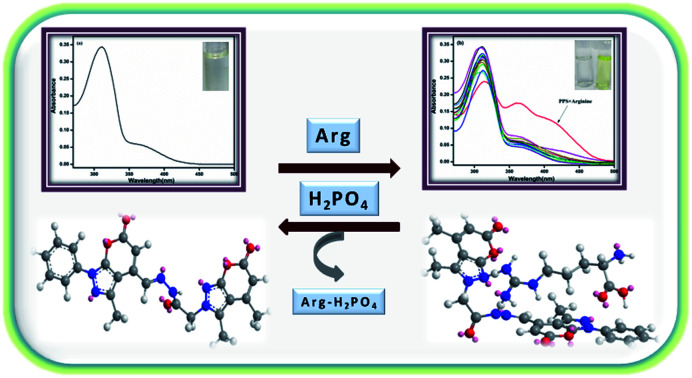

## 1. Introduction

Amino acids form the basic units of biological macromolecular proteins. Referred to as the building blocks of life, amino acids are placed into three different groups based on their requirement in our bodies. The amino acids that are naturally produced by our bodies and are not required in our daily diet are called non-essential amino acids. Essential amino acids are those which cannot be produced by our bodies and are required in our daily diets. However, there are a few amino acids that are essential in certain times of stress or illness as they are required at higher levels than produced by the body. Therefore, these amino acids are called conditionally essential amino acids. Out of the 20 amino acids, arginine is the most alkaline amino acid which is conditionally essential and has the highest isoelectric point (pI = 10.76). It plays a vital role in various biological functions such as the immune system, energizing protein regeneration and promoting the transportation of amino acids.^1^ It also promotes wound healing, detoxification of ammonia from the body, cell division and insulin secretion.^2–4^ In our bodies, arginine acts as a precursor for the synthesis of several biomolecules. It is an immediate precursor in the biosynthesis of nitric oxide, ornithine, agmantine and urea.^5,6^ Due to its participation in various biological processes; too much or too little presence of arginine or arginine derivatives might lead to different health problems.^7^ Therefore, developing viable methods for the detection of arginine has become an emerging area of research.^8–10^ Multiple methods for the determination of arginine have currently been reported in the literature, such as electrophoresis, fluorescence spectroscopy, high-performance liquid chromatography and molecular recognition technology.^11–14^ However, most of these techniques require complicated laboratory strategies and possess low selectivity which limits their utility. Hence, developing highly selective, fast responsive, inexpensive and simple methods for the recognition of arginine is desirable. Unlike fluorescence chemosensors, which require fluorescence as well as UV-vis apparatus, colorimetric chemosensors are an absolute approach owing to the convenient, sensitive and naked-eye detection ability of analytes based on a visible change in color and a change in absorption spectra.^15–17^

Polyfunctionalized fused heterocyclic compounds such as pyranopyrazoles and their derivatives have been highly emphasized in organic chemistry because of their structural resemblance to biologically active coumarin. The benzene ring of coumarin is substituted by a bioactive pyrazole ring to form pyranopyrazole, which is widely used as a parent compound to synthesize different natural products with biological significance.^18^ Like coumarins, pyranopyrazoles are also found to have anticancer,^19^ antimicrobial,^20^ anti-inflammatory^21^ and analgesic^22^ properties. A number of coumarin derivatives have been synthesized and used for the selective recognition of ions^23,24^ and neutral species.^25,26^ However, although a variety of pyranopyrazole derivatives have been synthesized, their utility as chemosensors has rarely been explored. To the best of our knowledge, we were the first group to develop a pyranopyrazole-based colorimetric sensor for the recognition of Fe^3+^ ions.^27^ In the present work, we have aimed at developing a novel Schiff base linked pyranopyrazole derivative (PPS) through a convenient synthetic route in good yield that acts as a potential colorimetric chemosensor for arginine.

## 2. Experimental

### 2.1 Materials and methods

The reagents and the solvents used for the synthesis and UV-vis studies were of analytical and spectroscopic grade. Phenyl hydrazine, hydrazine hydrate, ethyl 2-chloroacetate and ethyl acetoacetate were purchased from Spectrochem Pvt. Ltd and were used without further purification. All the amino acids used in UV-vis studies were purchased from Sigma Aldrich and Spectrochem Pvt Ltd. ^1^H and ^13^C NMR were recorded at room temperature on a JNM-EXCP 400 (JEOL, USA) spectrometer using TMS as an internal standard and the chemical shifts are reported in parts per million (ppm). FTIR data was recorded using a Shimadzu IR Affinity 1S Spectrophotometer. The crude products were purified using column chromatography (silica gel 100–200 mesh) and methanol–chloroform as the solvent system. UV-vis experiments were performed on a Carey Series UV-vis spectrophotometer using a 1 cm quartz cuvette. All experiments were performed in compliance with the policy statement on ethical considerations involved in research on human subjects (ICMR, 1980). These experiments were approved by the departmental research committee of University of Delhi. The blood sample of a healthy volunteer was procured from a local pathology lab and arginine granule supplements were commercially available in the market. The donor provided informed consent in accordance with the guidelines of the institution.

### 2.2 Synthesis of ethyl 2-(3,4-dimethyl-6-oxopyrano[2,3-*c*]pyrazol-2(6*H*)-yl) acetate (2)

**Scheme 1:**
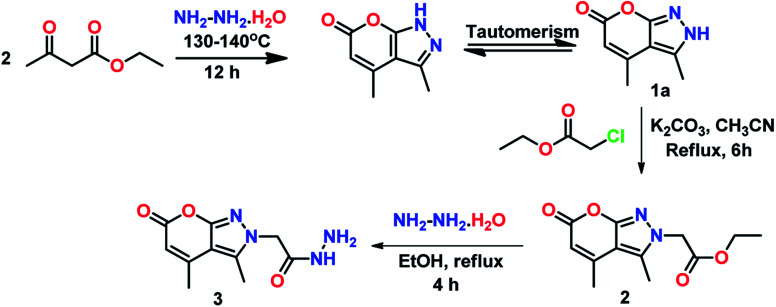
Synthetic route to compounds 2 & 3.

Anhydrous K_2_CO_3_ (2 mmol) was added to a solution of 3,4-dimethylpyrano[2,3-*c*]pyrazol-6(2*H*)-one (1a) (5 g, 1 mmol) in acetonitrile. To this ethyl 2-chloroacetate (2 mmol) was added drop-wise with constant stirring (Scheme 1). The reaction mixture was refluxed and the progress of the reaction was monitored through TLC. After completion of the reaction (6 h), the reaction mixture was allowed to cool to room temperature and then filtered to remove the solid precipitate. The filtrate obtained was concentrated under reduced pressure. The resultant crude product was recrystallized from ethanol as a white solid in 75% yield. The melting point was found to be 145–147 °C.

^1^H NMR (400 MHz, DMSO-d_6_): *δ* 5.88 (s, 1H), 5.12 (s, 2H), 4.17 (q, *J* = 7.1 Hz, 2H), 2.48 (s, 3H), 2.40 (s, 3H), 1.22 (t, *J* = 7.1 Hz, 3H). ^13^C NMR (100 MHz, DMSO-*d*_6_): *δ* 168.0, 161.1, 158.2, 152.6, 138.8, 108.2, 102.0, 61.9, 50.9, 19.2, 14.5, 11.4. HRMS (*m*/*z*): calculated for C_12_H_14_N_2_O_4_ [M + H]^+^ 251.0987; found 251.0980.

### 2.3 Synthesis of 2-(3,4-dimethyl-6-oxopyrano[2,3-*c*]pyrazol-2(6*H*)-yl) acetohydrazide (3)

To a solution of 2 (3 g, 1 mmol) in ethanol, hydrazine hydrate (2 mmol) was added and the mixture was heated under reflux conditions (Scheme 1). The reactant was consumed completely within 4 h, as monitored by TLC. After cooling in ice conditions, the resultant precipitates were filtered and washed with cold ethanol to obtain 3 as a white solid in 87% yield and melting point range was 282–284 °C.

^1^H NMR (400 MHz, DMSO-*d*_6_) *δ* 9.34 (s, 1H), 5.82 (s, 1H), 4.72 (s, 2H), 4.30 (s, 2H), 2.45 (s, 3H), 2.35 (d, *J* = 1.2 Hz, 3H). ^13^C NMR (100 MHz, DMSO-*d*_6_) *δ* 165.8, 161.2, 158.1, 152.7, 107.8, 101.9, 50.8, 19.2, 11.7. HRMS (*m*/*z*): calculated for C_10_H_12_N_4_O_3_ [M + H]^+^ 237.0943; found 237.0984.

### 2.4 Synthesis of 2-(3,4-dimethyl-6-oxopyrano[2,3-*c*]pyrazol-2(6*H*)-yl)-*N*′-((3-methyl-6-oxo-1-phenyl-1,6-dihydropyrano[2,3-*c*]pyrazol-4-yl)methylene) acetohydrazide (PPS)

**Scheme 2:**
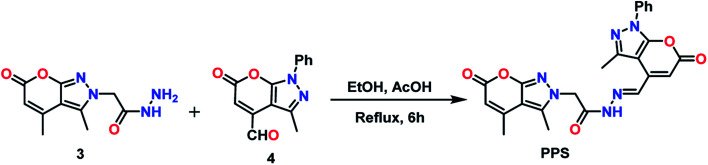
Synthetic route to PPS.

As shown in Scheme 2, 3 (200 mg, 2 mmol) and 4 (1 mmol) were mixed in ethanol by subsequently adding few drops of glacial acetic acid. The reaction mixture was then refluxed and product formation was observed through TLC. The reaction was complete in 6 h and the reaction mixture was allowed to cool under ice conditions. The precipitates then obtained were filtered and washed with cold ethanol to obtain a pale green solid as the crude product. The product was then purified using column chromatography (CHCl_3_–MeOH, 9 : 1) to procure PPS as a pale yellow solid in 70% yield. The melting point of PPS was found to be 292–294 °C.

^1^H NMR (400 MHz, DMSO-*d*_6_): *δ* 12.29 (s, 1H), 8.27 (s, 1H), 7.77–7.42 (m, 5H), 6.43 (s, 1H), 5.86 (s, 1H), 5.48 (s, 1H), 5.07 (s, 1H), 2.54 (s, 3H), 2.48 (s, 3H), 2.40 (s, 3H). ^13^C NMR (100 MHz, DMSO-*d*_6_) *δ* 168.7, 161.1, 159.3, 158.0, 152.5, 151.2, 145.6, 144.3, 139.1, 138.4, 136.8, 130.0, 127.9, 121.5, 121.2, 107.9, 102.0, 99.8, 51.0, 19.1, 17.4, 15.9, 11.5. HRMS (*m*/*z*): calculated for C_24_H_20_N_6_O_5_ [M + H]^+^ 473.1529; found 473.1579.

### 2.5 UV-vis studies

To explore the interactions of PPS with various amino acids, UV-vis experiments were performed. A stock solution of PPS (10 mM) was prepared in DMSO and stock solutions of 100 mM of the amino acids alanine, valine, cystine, glycine, glutathione, homocystine, histidine, leucine, lysine, phenylalanine, proline and serine were prepared in double-distilled water. The stock solutions of amino acids were stored at 4 °C and used within ten days of preparation. To carry out the binding studies, 10 μM of PPS solution was prepared in DMSO–PBS (8 : 2 v/v, 10 mM, pH = 7.4). 30 μL of stock solution of an amino acid was added to 2.97 mL of PPS (10 μM) in a quartz cuvette. The absorption spectra of PPS were recorded in the absence and presence of various amino acids.

### 2.6 Selectivity studies

To analyze the interference by other amino acids in the recognition of arginine, competitive binding studies were performed. To achieve this, the colorimetric and spectral changes of PPS–arginine (10 : 5 μM) were observed in the presence of 5 μM of other amino acids.^28^

### 2.7 Binding stoichiometry analysis & limit of detection

The binding affinity of PPS with arginine was examined through absorption titration experiments by successive additions of arginine (0–20 μM with an interval of 2 μM). The absorption spectra thus obtained were used to construct a Benesi–Hildebrand plot of 1/(*A* − *A*_0_) *vs.* 1/[Arg]. The binding constant was calculated from the slope of the B–H plot from eqn (1):1
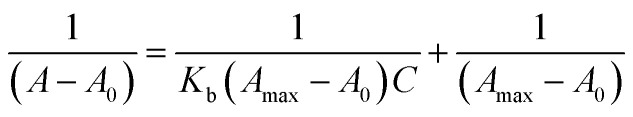


Here *A*_0_ and *A* are the absorption intensities of PPS in the absence and presence of arginine, respectively. *C* is the concentration of arginine, *K*_b_ is the binding constant and *A*_max_ is the maximum absorption intensity observed after the addition of arginine.

To find the binding stoichiometry, stock solutions of PPS (10 μM) and arginine (10 μM) were prepared in DMSO-PBS (8 : 2 v/v, 10 mM, pH = 7.4) and double-distilled water, respectively. Then 0.3–3 mL of PPS and 3–0.3 mL of arginine were added respectively to keep the total volume in the quartz cuvette at 3 mL for recording the absorption spectra. The Job's plot was obtained by plotting a graph between mole fraction of PPS (*X*_PPS_) and (*A*_0_ − *A*) × *X*_PPS_.^29^

The limit of detection of PPS for arginine was calculated using eqn (2):2
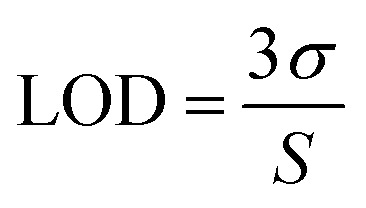


Ten replicate absorption measurements were taken to calculate standard deviation (*σ*) and *S* is the slope of the calibration curve.

### 2.8 Computational methods

The binding interactions of the synthesized ligand with amino acid arginine were studied theoretically based on the Density Functional Theory (DFT) method using the Gaussian 09 program at the B3LYP/6-311++G(d,p) level of theory.^30^ A TD-DFT study was carried out by employing a hybrid B3LYP and 6-311+G basis set in Gaussian 09. Careful analysis of the vibrational frequencies confirms that all the stationary points in the way of geometrical optimization correspond to local minima. The sum of electronic energy (*ε*_o_) and thermal free energy (*G*_corr_) were used in the computation of Δ*G* values. The integral equation formalism polarized continuum model (IEFPCM) was employed for single point time-dependent DFT (TD-DFT) calculations on the optimized geometry in order to account for the solvent effect of DMSO.^31^

### 2.9 Hemolysis assay

The blood compatibility of PPS was studied using a hemolysis assay. Briefly, blood was collected from a healthy voluntary human donor in a heparinized tube, centrifuged for 5 min at 4 °C, and the pellet (cells) washed 3 times with 1× PBS (pH = 7.4) as described previously.^32^ The human RBCs were treated with different concentrations of PPS (10, 25, 50, and 100 μM) for 1 h at 37 °C. The amount of hemoglobin released was analyzed with a microplate reader at 540 nm and the percentage of hemolysis was calculated using eqn (3):3
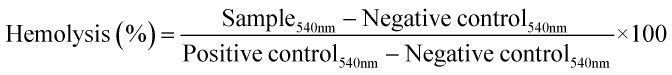


1% Triton X-100 was used as a positive control which achieved complete hemolysis (100%) and 1× PBS (pH = 7.4) served as the negative control. All the experiments were performed in triplicate. After incubation, the RBCs were again isolated by centrifugation, resuspended in 1× PBS, mounted on a wet slide and examined using a bright field light microscope.

## 3. Results and discussions

### 3.1 Synthesis

The final product PPS was synthesized by mixing ethanolic solutions of 3 and 4 and adding a few drops of acetic acid to the resulting reaction mixture. This reaction mixture was refluxed for 6 h to obtain PPS in 70% yield. The precursors of the final compound PPS were synthesized from their parent molecules 1a and 1b which were prepared using a modified Pechmann condensation reaction of ethyl acetoacetate with hydrazines at 130–140 °C through *in situ* generation of pyrazolone (Scheme S1†). Reactant 4 was synthesized by oxidation of 3,4-dimethyl-1-phenylpyrano[2,3-*c*]pyrazol-6(1*H*)-one (1b) in the presence of selenium dioxide and 1,4-dioxane as solvent, under reflux conditions, as already reported by our research group (Scheme S2†).^33^ The formation of all the synthesized compounds was confirmed by ^1^H NMR, ^13^C NMR, FT-IR and mass spectroscopy.

### 3.2 Colorimetric recognition of arginine

**Fig. 1:**
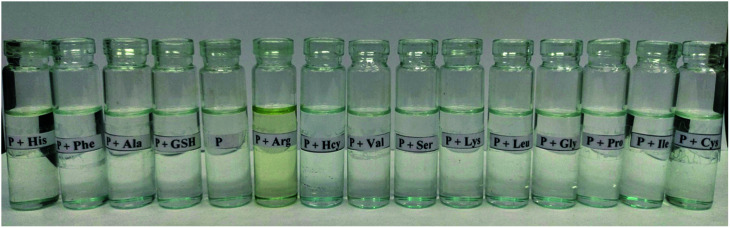
Colorimetric variations in PPS (10 μM) on addition of 10 equiv. of different amino acids.

The colorimetric sensing of ligand PPS was investigated by the addition of thirteen different amino acids to solutions of PPS. Only in the case of the addition of arginine was an immediate remarkable change from colorless to yellow observed while no color changes were observed on the addition of the other amino acids (Fig. 1).

### 3.3 Absorption studies

**Fig. 2:**
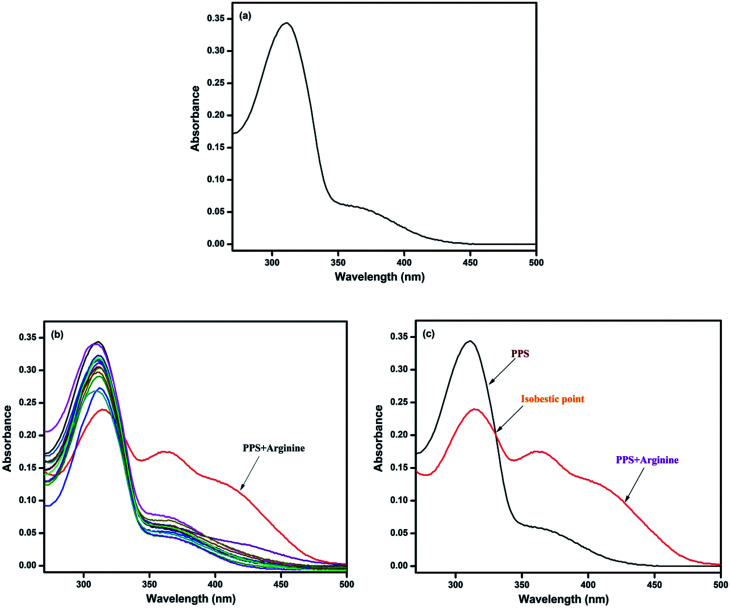
(a) Absorption spectrum of PPS (10 μM) in DMSO–PBS (8 : 2 v/v, 10 mM, pH = 7.4). (b) Spectral changes in PPS on addition of 10 equiv. of different amino acids. (c) Absorption spectra of PPS and PPS + arginine.

The UV-vis absorption spectrum of PPS (10 μM) recorded in DMSO–PBS (8 : 2 v/v, 10 mM, pH = 7.4) displayed a broad band in the range of 270–500 nm with an absorption maximum at 310 nm (Fig. 2a) which might be attributed to the π–π* or n–π* transitions within the ligand. When 10 equiv. of amino acids, such as alanine, valine, cystine, glycine, glutathione, homocystine, histidine, leucine, lysine, phenylalanine, proline and serine, were added to the stock solution of PPS, no significant changes were observed in its absorption spectra. However, the addition of arginine to PPS resulted in a prominent change in the absorption spectra, as shown in Fig. 2(b). It can be seen in Fig. 2(c) that in the presence of arginine, the intensity of the charge transfer band of PPS centered at 310 nm prominently decreased with a red shift of 5 nm while the absorption intensity at 363 nm increased. Furthermore, a new band appeared at 410 nm with an isobestic point at 330 nm, suggesting the formation of a stable complex between PPS and arginine.

### 3.4 Selectivity studies

**Fig. 3:**
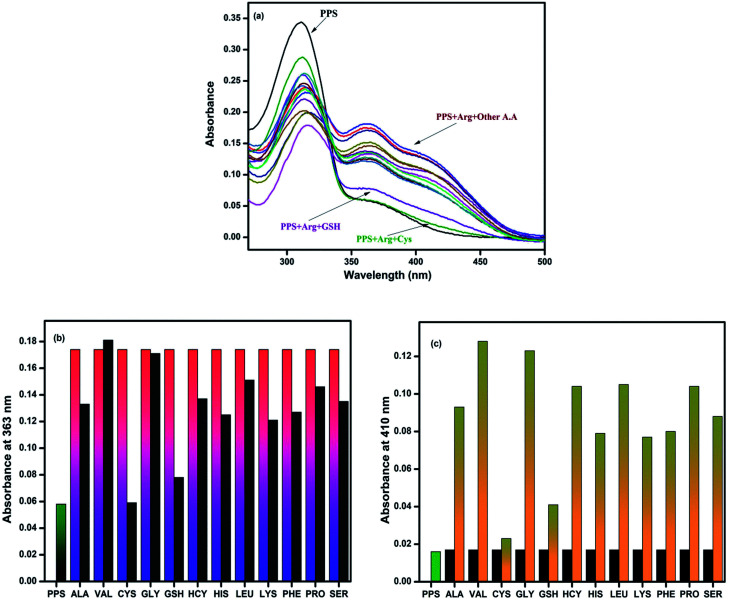
(a) Absorption spectral changes of PPS in DMSO–PBS (8 : 2 v/v, 10 mM, pH = 7.4) on addition of different amino acids in the presence of arginine. (b) Absorbance data for PPS and amino acids at 363 nm. (c) Absorbance data for PPS and amino acids at 410 nm.

To assess the selectivity of PPS for arginine in the presence of a complex background of other co-existing competitive amino acids, an interference experiment was carried out, as shown in Fig. 3. The experiment revealed in Fig. 3(a) that the absorption spectra of PPS containing arginine (5 equiv.) showed similar patterns in the presence of other amino acids (5 equiv.). However, slight interference was observed with CYS and GSH. Based on the response towards the interfering samples, the chemosensor PPS showed selectivity for arginine in the presence of other amino acids except CYS and GSH under the same conditions (Fig. 3(b) and (c)).

### 3.5 Binding stoichiometry analysis

**Fig. 4:**
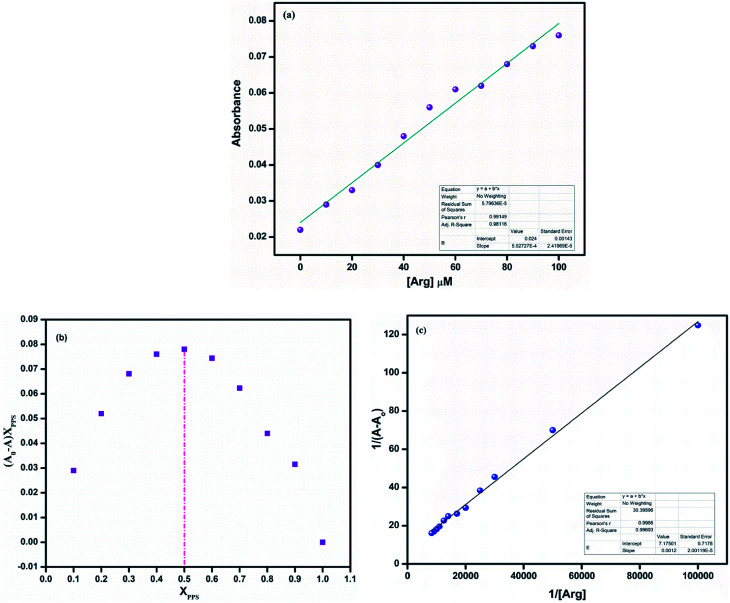
(a) Absorption intensity of PPS (10 μM) on subsequent addition of arginine (0–100 μM). (b) Job's plot of PPS with arginine in DMSO–PBS (8 : 2 v/v, 10 mM, pH = 7.4). (c) B–H plot of PPS with arginine in DMSO–PBS (8 : 2 v/v, 10 mM, pH = 7.4).

To get further perceptions on the interaction between the ligand PPS and arginine, titration experiments were carried out in DMSO–PBS (8 : 2 v/v, 10 mM, pH = 7.4). On successive addition of arginine to PPS, it could be observed that the absorption intensity at 410 nm gradually increased (Fig. 4(a)).

Furthermore, to recognize the coordination of PPS with arginine, the binding constant and the binding stoichiometry were determined using a Job's plot and Benesi–Hildebrand plot, respectively. In the Job's plot (Fig. 4(b)), it could be observed that the maximum was achieved when the molar ratio of the chemosensor was 0.5. This suggested 1 : 1 binding stoichiometry between PPS and arginine. The B–H plot of 1/(*A* − *A*_0_) *vs.* 1/[Arg] showed linearity with *R*^2^ = 0.99693, which also confirmed the 1 : 1 binding stoichiometry of the complex (Fig. 4(c)). The binding/association constant of PPS for arginine was found to be 1.57 × 10^4^ M^−1^.

### 3.6 Limit of detection & time response

**Fig. 5:**
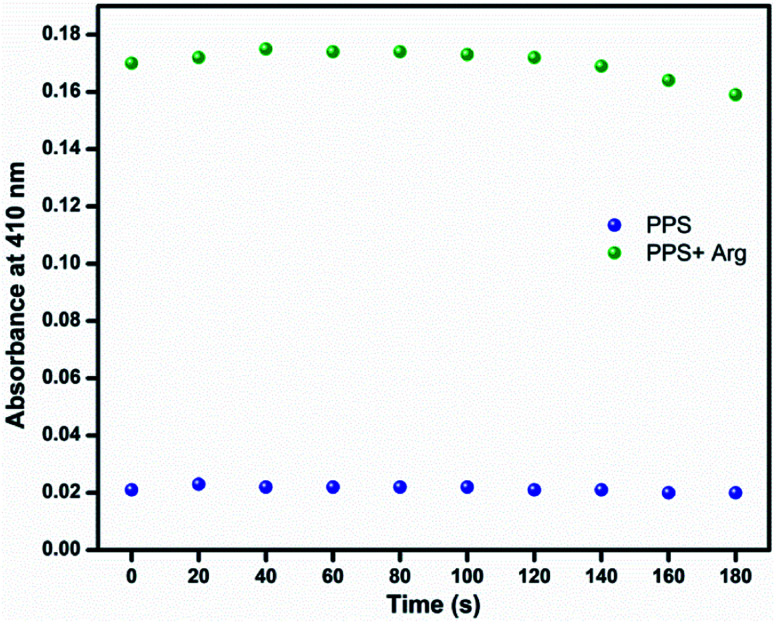
Time-dependent absorption response of PPS (10 μM, DMSO–PBS, pH = 7.4, 8 : 2, v/v) at 410 nm in the presence of 1 equiv. of arginine.

The limit of detection was calculated to be 1.8 × 10^−5^ M. This implied that the ligand PPS could be used for quantitative detection of arginine even at low concentrations. The absorbance of PPS (10 μM) at 410 nm changed immediately when 1 equiv. of arginine was added and remained constant afterwards (Fig. 5). The ultrafast sensing of PPS made it suitable for the real-time detection of arginine.

### 3.7 Plausible sensing mechanism

The binding modes of PPS with arginine were further studied through ^1^H NMR titration experiments. Upon addition of 0.5 equiv. of arginine to PPS in DMSO-*d*_6_, the peak at 12.29 ppm was almost completely suppressed. However, no other changes were observed in the NMR spectrum (Fig. S7†). This suggested that the binding of arginine to PPS occurred through the –NH proton of the Schiff base unit present in PPS. The plausible binding mechanism is shown in Scheme S3.† The mechanism draws further support from the observations from computational studies in the following section.

### 3.8 Computational studies

**Fig. 6:**
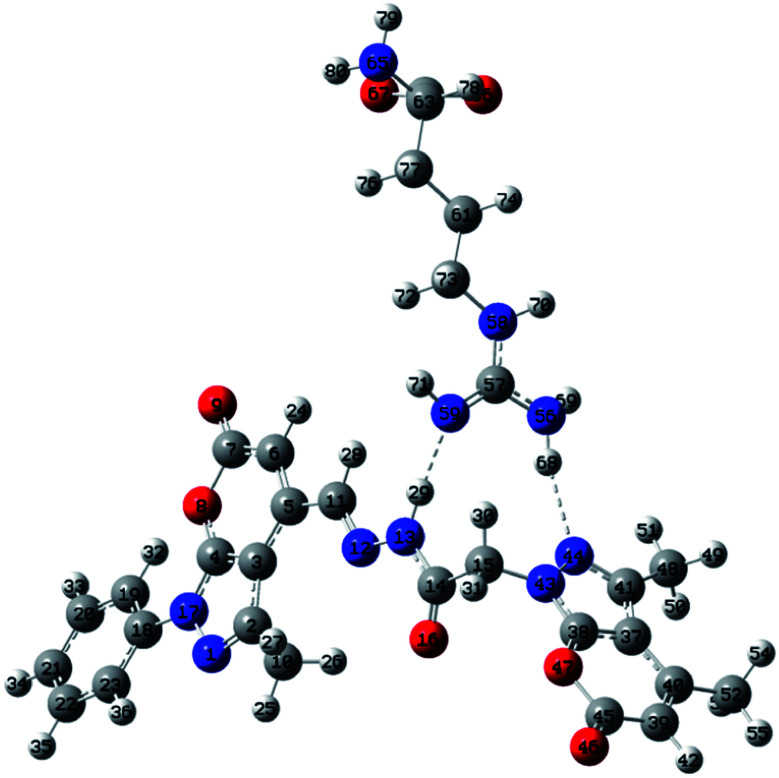
Most stable configuration of PPS–arginine complex.

DFT calculations were performed with the objective of gaining an insight into the binding interaction of PPS with arginine. A strong binding interaction is indicated in the PPS–arginine complex exhibiting free energy, Δ*G* = −13.6 kcal mol^−1^ for the formation of the complex *via* H-bonding. As per the energetic considerations, it is interesting to note that the ligand undergoes a conformational change, from PPS (a) to PPS (b) in the structure of the molecule, thereby allowing for efficient binding to arginine at two different sites in order to form a more stable complex structure (Fig. S8†). It is evident from Fig. 6 that hydrogen bonds of the type N–H–N have been formed at two different binding sites that are represented as N_59_–H_29_–N_13_ (site 1) and N_44_–H_68_–N_56_ (site 2) with H-bond distances of 1.87 Å and 2.17 Å, respectively. Natural bond orbital (NBO) analysis was also performed to investigate the relative strength of these binding interactions in the PPS–arginine complex. The stabilization energies for intermolecular interactions, between the lone-pair electrons of the proton acceptor and antibonding orbitals of the proton donor, obtained using second-order perturbation theory as implemented in the NBO analysis, account for stronger interaction at site 1 (7.36 kcal mol^−1^) compared to site 2 (3.77 kcal mol^−1^). These trends in stabilization energies are in accord with the observed H-bond distances at the two different sites in the complex.

**Fig. 7:**
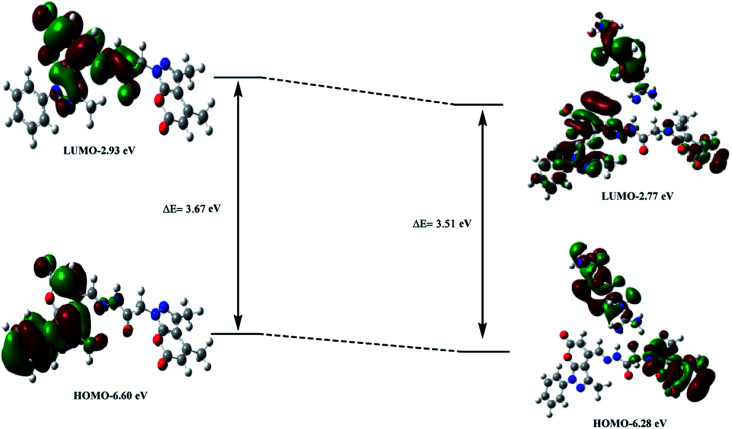
The HOMO–LUMO gap calculated by DFT studies.

The TD-DFT computations were aimed at achieving better insight into and a thorough understanding of the nature of the transitions in the UV-vis spectra. A careful analysis of the electronic states of PPS and PPS–arginine suggested that a set of closely populated occupied MOs (Fig. S9 & S10†) are involved in electronic excitation (at *λ*_max_) which are primarily represented by electronic transitions from HOMO-5 to LUMO. The electron density in the PPS–arginine complex in HOMO-5 is primarily centered on arginine, which indicates a charge transfer from arginine to PPS in the complex. The HOMO and LUMO orbitals corresponding to PPS and the PPS–arginine complex have been depicted in Fig. 7. The calculated energy gap (Δ*E*) between HOMO and LUMO was found to be lower than that of the free ligand, illustrating the occurrence of a red shift on the addition of arginine to PPS.

The results of the TD-DFT study clearly revealed a red shift in the absorption maximum of the ligand from 303 nm to 319 nm, upon complexation with arginine. Moreover, the oscillator strength of the specified primary transition was also observed to decrease from 0.9423 to 0.1814, which accounts for the decrease in absorption intensity at *λ*_max_ in the UV-vis spectra of the ligand upon complexation. These results thus exhibit reasonable agreement with the experimentally observed UV-vis spectra. It may be noted that the TD-DFT computations tend to overestimate or underestimate energies of the excited states^34,35^ owing to the local nature and asymptotic behavior of the approximate functional with special reference to the exchange correlation functional in the case of charge transfer transitions. Consequently, we observe deviations of *λ*_max_ values in theoretically obtained UV-vis spectra in comparison to the experimental spectra (Table S2†).

### 3.9 Reversibility

**Fig. 8:**
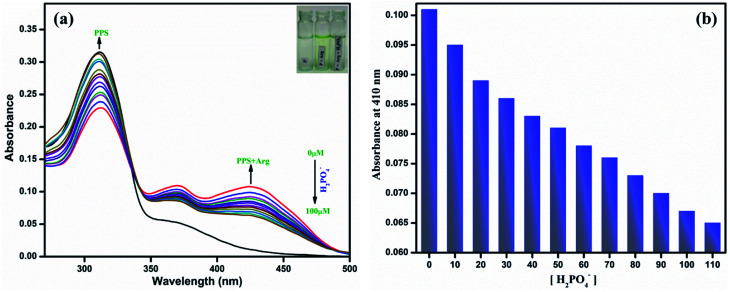
(a) Absorption spectra of PPS (10 μM, DMSO–PBS, pH = 7.4, 8 : 2, v/v) with arginine (100 μM) and successive additions of KH_2_PO_4_ (0–100 μM). (b) Absorbance of PPS–arginine (10 : 100 μM, DMSO–PBS, pH = 7.4, 8 : 2, v/v) at 410 nm *vs.* increasing concentration of H_2_PO_4_^−^.

The most important aspect for a sensor to be employed for practical applications is its reversibility and recyclability. We have seen that PPS has proved to be sensitive towards arginine. However, there have been literature reports^36^ that arginine has the ability to bind phosphates through electrostatic interactions. Therefore, to examine the reversibility of PPS–arginine, we selected H_2_PO_4_^−^ ions. It was observed that on successive additions of H_2_PO_4_^−^, the absorption intensity of the band due to PPS–arginine (at 410 nm) decreased while that due to PPS alone (310 nm) started to increase (Fig. 8(a)). The absorption intensity at 410 nm has also been plotted against increasing concentration of H_2_PO_4_^−^ (Fig. 8(b)). Moreover, a naked-eye color change from yellow to colorless was also noticed, which further supported the reversibility. Hence, PPS exhibited good reversibility and has the potential to be used in practical applications.

### 3.10 Hemolysis study

**Fig. 9:**
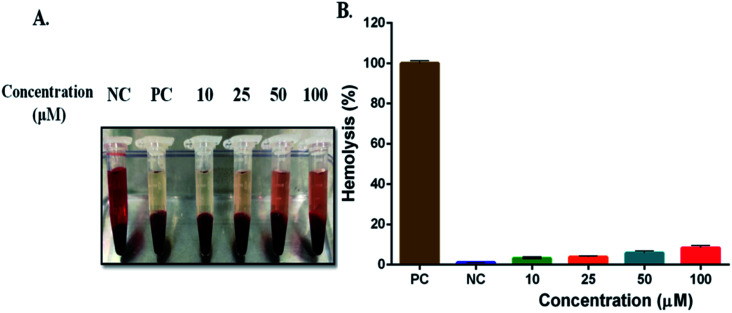
Hemolysis assay. (A) RBCs were incubated with PPS at different concentrations for 1 h at 37 °C. Triton X-100 (1%) and normal saline were used as positive and negative controls (PC & NC), respectively. (B) Quantitative estimation of RBCs membrane damage and haemoglobulin release.

The *in vitro* blood biocompatibility and hemolysis of PPS was studied at various concentrations (10, 25, 50, and 100 μM). After incubation, human RBCs remain unaffected by all concentrations of PPS while complete membrane damage to RBCs (hemolysis) was observed in 1% Triton X-100 (positive control), as shown in Fig. 9. No hemolysis was observed in saline (negative control) whereas PPS showed 3–8% hemolysis after incubation for 1 h at 37 °C which was not significant, indicating its blood compatibility nature.

### 3.11 Real sample analysis

#### 3.11.1 Detection of arginine in arginine supplements

**Fig. 10:**
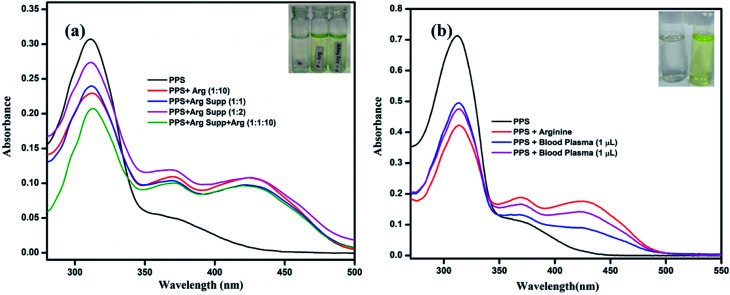
(a) Colorimetric detection of arginine by PPS in arginine supplements. (b) Colorimetric detection of arginine by PPS in human blood plasma.

To examine the practical utilization of the chemosensor PPS towards arginine, l-arginine granules were selected as a real sample. 250 mg of these granules were dissolved in 5 mL of distilled water and the solution was filtered. Two samples were prepared by adding 10 μL and 20 μL of this solution to a 3 mL solution of PPS (10 μM, DMSO–PBS, pH = 7.4, 8 : 2, v/v). Another sample was prepared by spiking 100 μM of arginine solution into a solution of PPS containing 10 μL of granule solution. The absorption spectra of these samples were recorded, as can be seen in Fig. 10(a). The absorption spectrum of PPS containing arginine granules showed a similar pattern to that of PPS–arginine. Therefore it can be inferred that PPS has the potential to be used in real sample analysis.

#### 3.11.2 Detection of arginine in blood plasma

To further examine the utility of PPS in humans, we were inspired to detect arginine in biological samples as well. In blood, low molecular weight arginine is present in the free state and is also bound to proteins in which it is present in higher percentages. For the present study to determine arginine in biological samples, we used blood plasma as a source. The additions of human blood plasma to PPS lead to a color change in the probe along with changes in the UV-vis absorption spectra. The color change of the solution from colorless to yellow was readily detectable by the naked eye. Also, the absorption spectra showed a decrease in the band intensity at 320 nm and the formation of a new band at 410 nm (Fig. 10(b)). These observations were the same as those in the experiments performed above. Therefore, it is worth mentioning that PPS is a potential chemosensor for the selective detection of arginine over other amino acids in human blood plasma.

## 4. Conclusions

In conclusion, a novel Schiff base PPS has been successfully synthesized by the condensation reaction of aldehyde and hydrazine derivatives of pyranopyrazole. The formation of PPS was confirmed through NMR, IR and mass spectrometry. PPS acted as a turn-on colorimetric sensor for the amino acid arginine in DMSO–PBS (8 : 2 v/v, 10 mM, pH = 7.4). Arginine formed a 1 : 1 complex with PPS with binding constant *K*_a_ = 8.57 × 10^4^ M^−1^, as evident from the Job's plot and Benesi–Hildebrand plot, respectively. Selectivity studies revealed that PPS acted as a selective chemosensor for arginine in the presence of other amino acids; however, interference was observed in the presence of Cys and GSH. The detection limit of PPS for arginine was found to be 1.8 × 10^−5^ M. The formation of a PPS–arginine complex was established using ^1^H NMR titration and DFT experiments. PPS–arginine formed “*in situ*” was found to show reversible absorption and colorimetric response towards H_2_PO_4_^−^. Moreover, PPS could efficiently detect arginine in arginine supplement granules and blood plasma. Therefore, it could be inferred that the chemosensor PPS acted as a potential sensor for arginine in real samples.

## Conflicts of interest

The authors declare no conflict of interest.

## Supplementary Material

RA-012-D2RA00091A-s001
