# Supplementary material for: Pyranopyrazole based Schiff base for rapid colorimetric detection of arginine in aqueous and real samples
Source: RSC Adv. 2022 Apr 19;12(19):11942–52. doi: 10.1039/d2ra00091a (PMC9017462; doi:10.1039/d2ra00091a)
Supplement: RA-012-D2RA00091A-s001 [file RA-012-D2RA00091A-s001.pdf]

Electronic Supplementary Material (ESI) for RSC Advances.
This journal is © The Royal Society of Chemistry 2022

Pyrano-pyrazole based Schiff Base for Rapid Colorimetric Detection of Arginine in aqueous and Real Samples

Contents

Scheme S1: Synthetic Route to precursors

Scheme S2: Synthetic Route to compound **4**

Figure S1: ^{1}H NMR spectrum of **2** in DMSO-d_6

Figure S2: ^{13}C NMR spectrum of **2** in DMSO-d_6

Figure S3: ^{1}H NMR spectrum of **3** in DMSO-d_6

Figure S4: ^{13}C NMR spectrum of **3** in DMSO-d_6

Figure S5: ^{1}H NMR spectrum of **PPS** in DMSO-d_6

Figure S6: ^{13}C NMR spectrum of **PPS** in DMSO-d_6

Figure S7: ^{1}H NMR titration experiment of **PPS** with different equiv. of Arginine in DMSO-d_6

Scheme S3: Plausible binding mechanism of complex formation between **PPS** and arginine.

Figure S8: Optimized geometries of **PPS** and **PPS**-Arginine

Figure S9: Pictorial representation of different MO's of PPS

Figure S10: Pictorial representation of different MO's of PPS-Arginine

Table S1: Parameters obtained from the TD-DFT calculations. (f = oscillator strength)

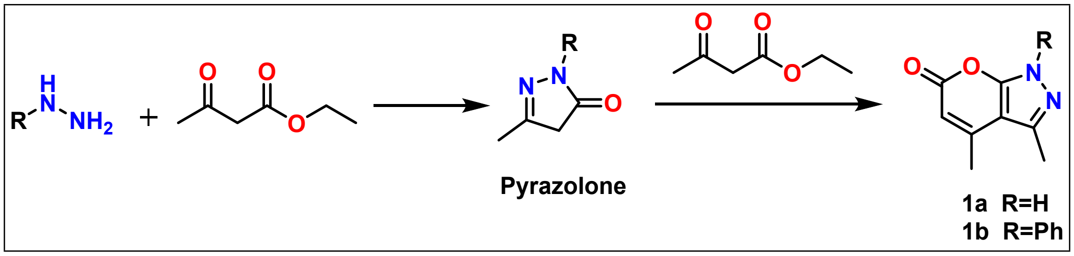

Scheme S1: Synthetic Route to precursors **1a** and **1b**

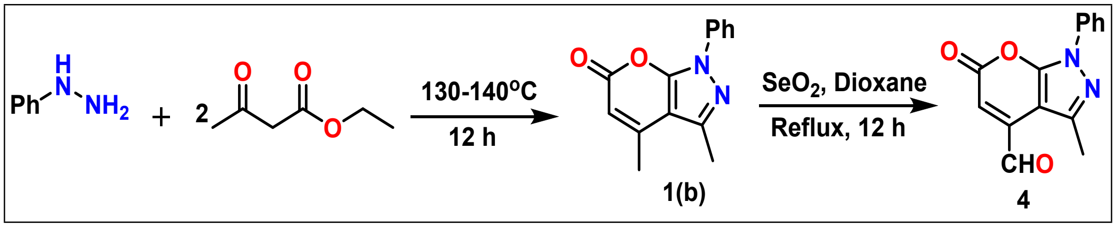

Scheme S2: Synthetic Route to compound **4**

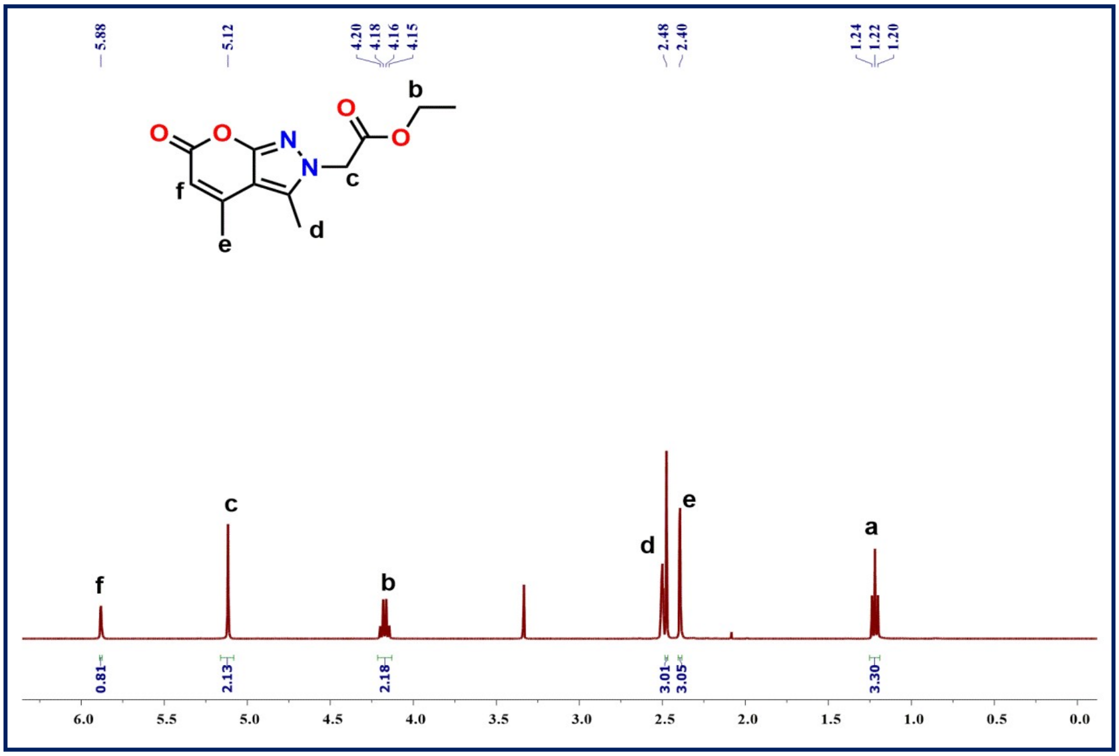

Figure S1: ^{1}H NMR of **2** in DMSO-d_6

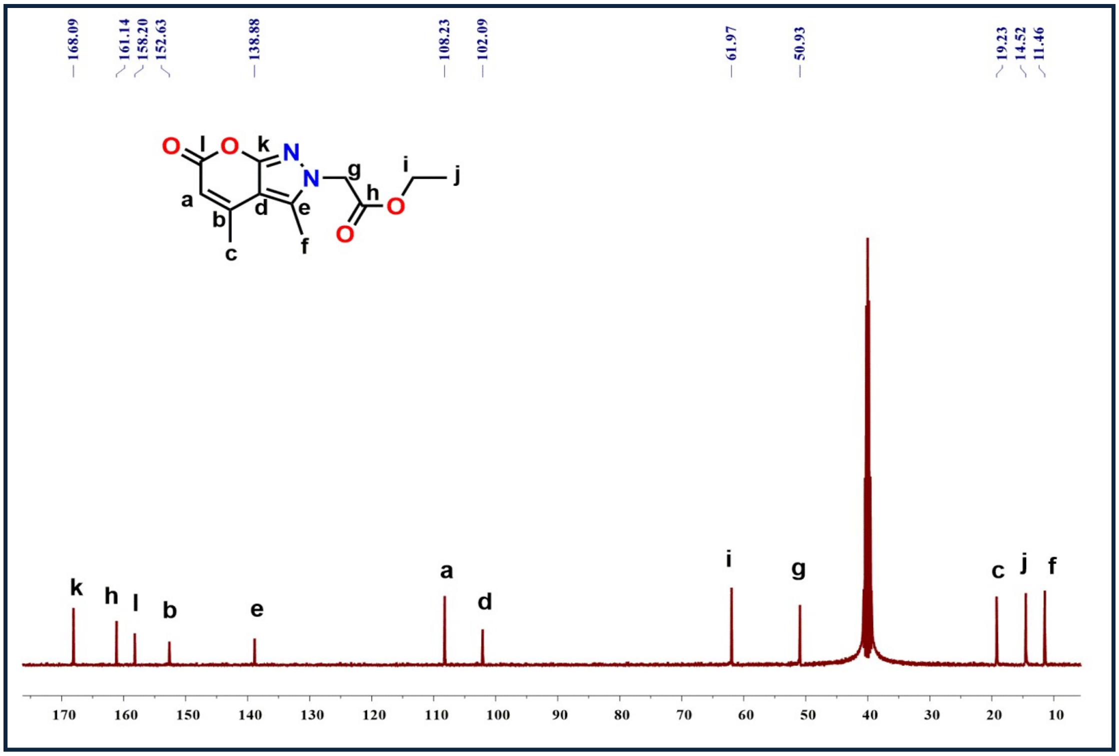

Figure S2: ^{13}C NMR of **2** in DMSO-d_6

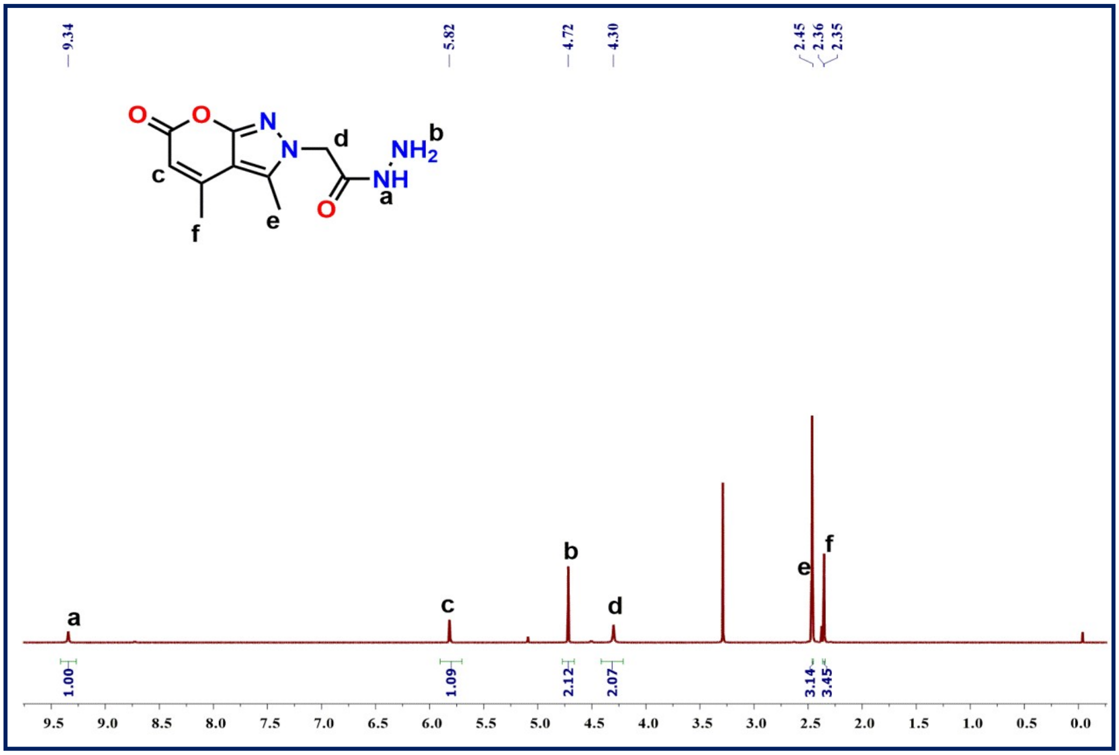

Figure S3: ^{1}H NMR of **3** in DMSO-d_6

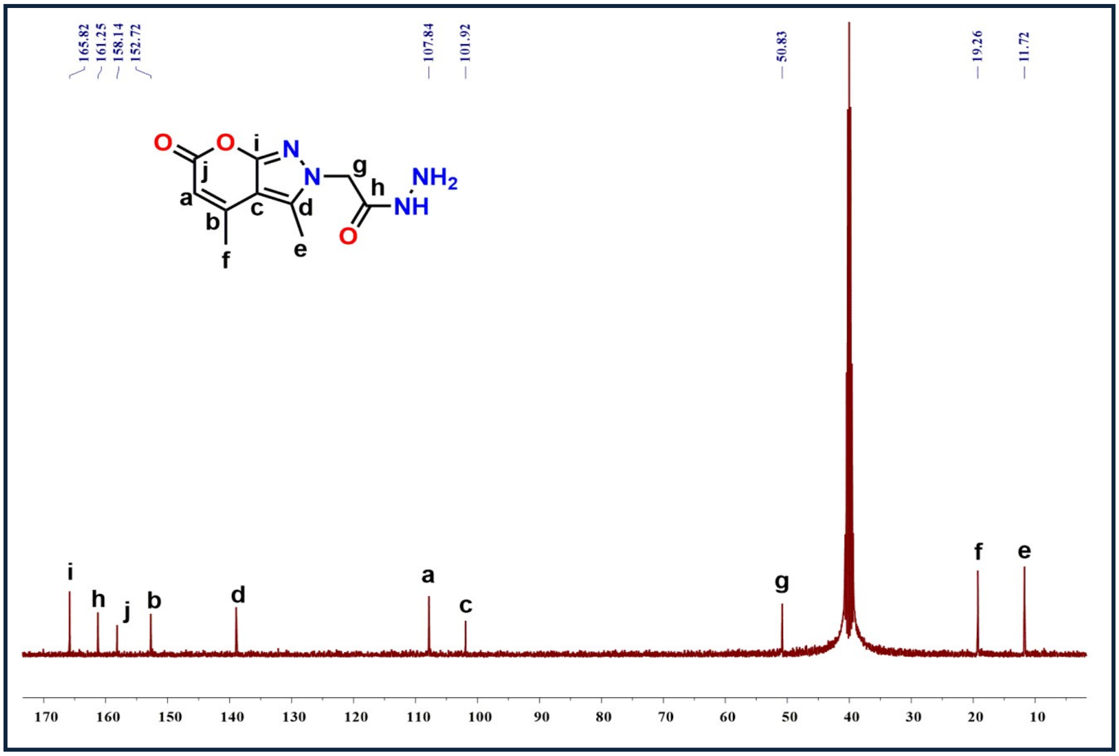

Figure S4: ^{13}C NMR of **3** in DMSO-d_6

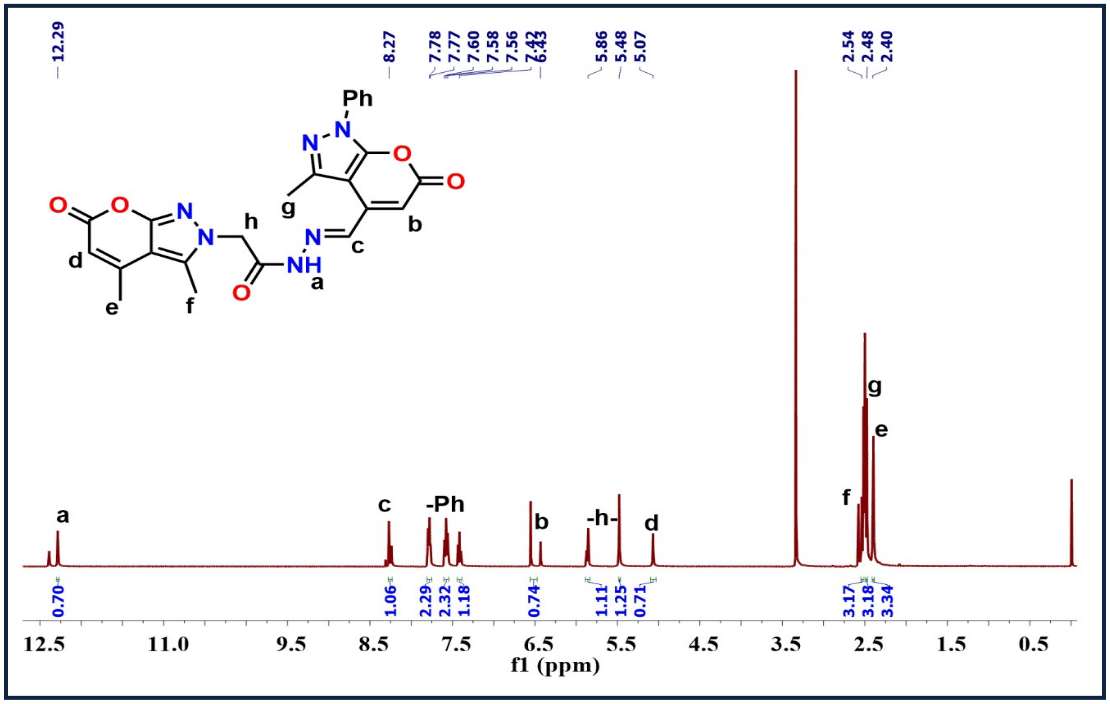

Figure S5: ^{1}H NMR spectrum of **PPS** in DMSO-d_6

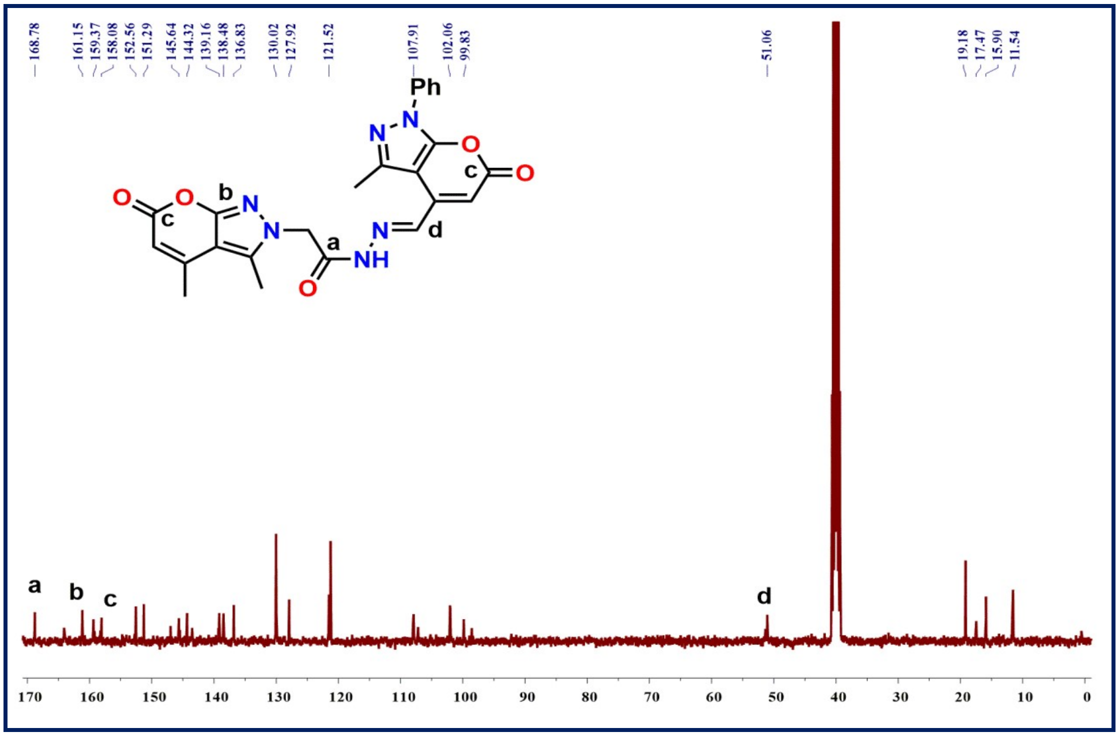

Figure S6: ^{13}C NMR spectrum of **PPS** in DMSO-d_6

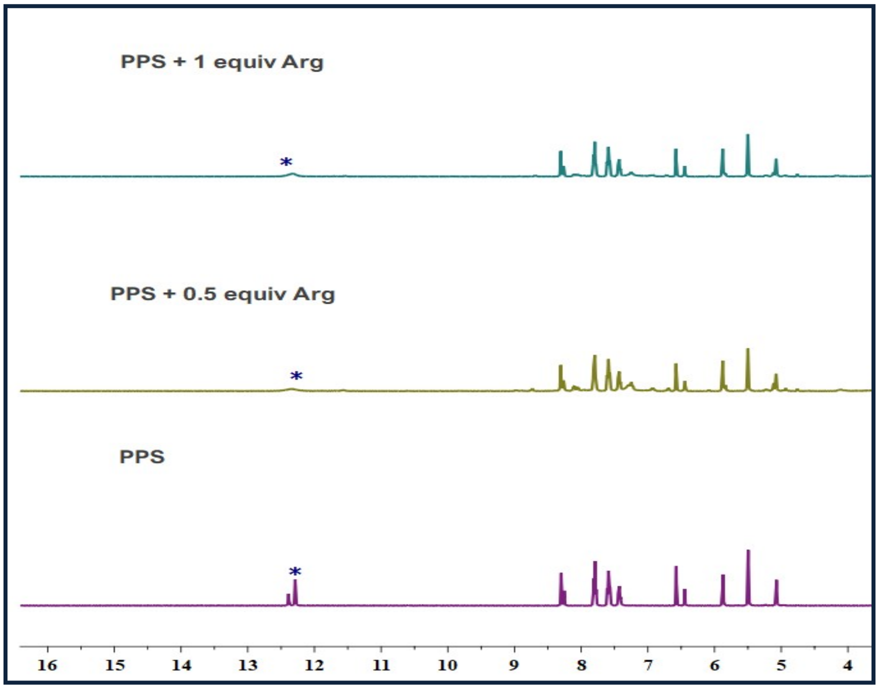

Figure S7: ^{1}H NMR titration experiment of **PPS** with different equiv. of Arginine in DMSO-d_6

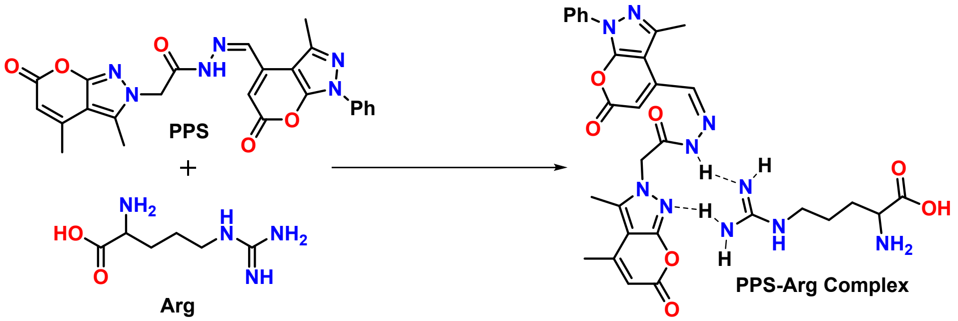

Scheme S3: Plausible binding mechanism of **PPS**-arginine complex formation

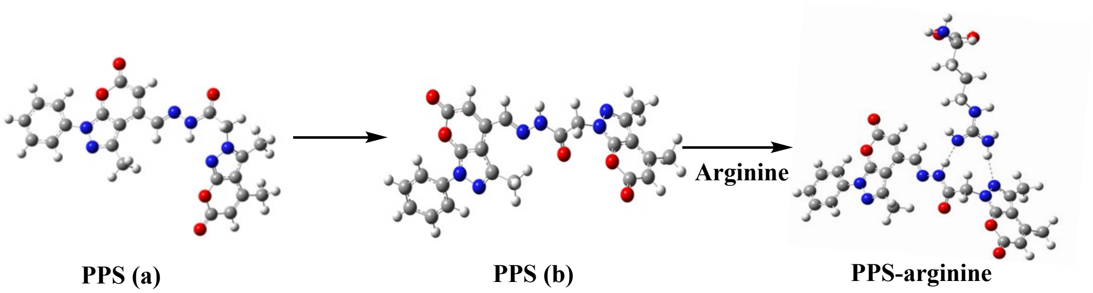

Figure S8: Optimized geometries of **PPS** and **PPS**-Arginine

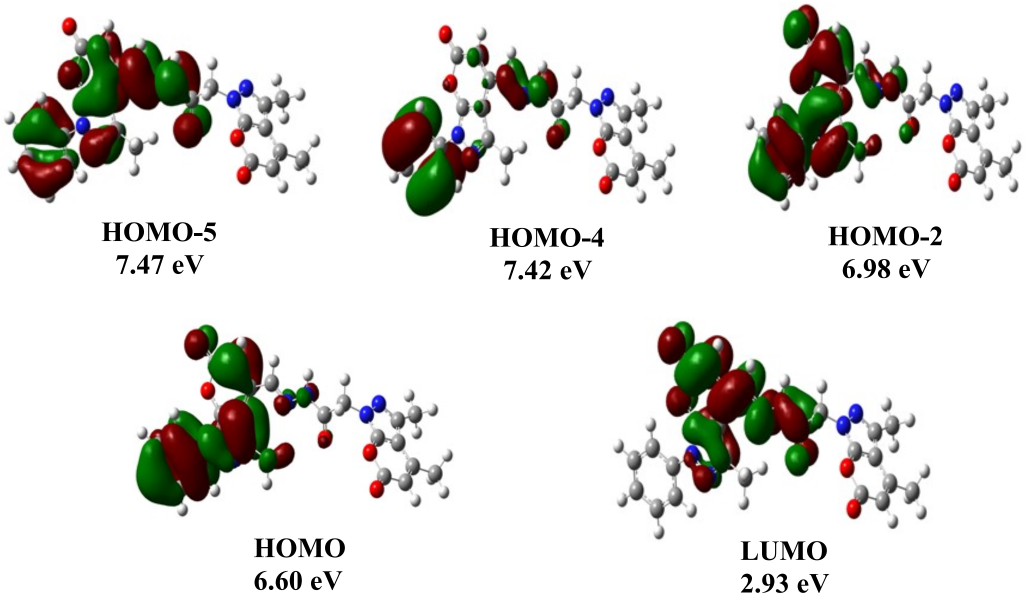

Figure S9: Pictorial representation of different MO's of **PPS**

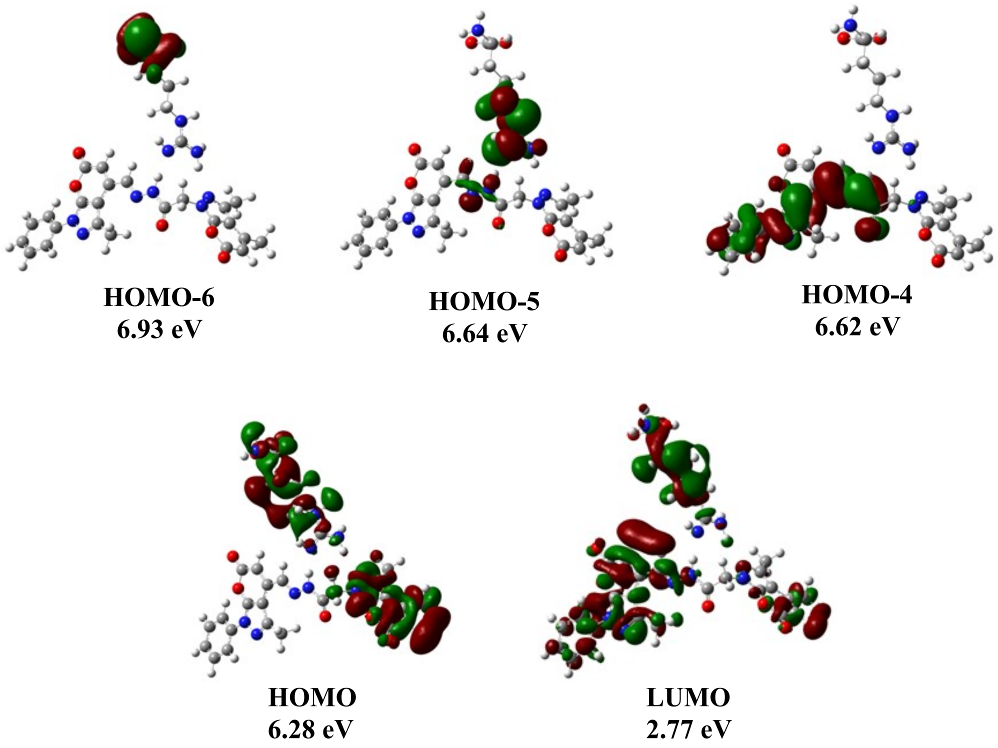

Figure S10: Pictorial representation of different MO's of **PPS-**Arginine

Table S1: Parameters obtained from the TD-DFT calculations. (f = oscillator strength)

	**Experimental λ_{max} Values**	**TD-DFT**		
		**Transition-1**	**Transition-2**	**Transition-3**
Ligand	310 nm	303.35 nm / 4.08 eV<br>HOMO-5 to LUMO<br>f = 0.9423	343.69 nm / 3.60 eV<br>HOMO-2 to LUMO<br>f = 0.1312	
Ligand-Arginine Complex	315 nm, 363 nm and 402 nm	319.04 nm / 3.89 eV<br>HOMO-5 to LUMO<br>f = 0.1814	349.56 nm / 3.54 eV<br>HOMO-2 to LUMO<br>f = 0.1381	382.04 nm / 3.25 eV<br>HOMO to LUMO<br>f = 0.0682
